# Quercetin in skin burn healing: mechanisms, advanced delivery systems, and translational perspectives

**DOI:** 10.3389/fbioe.2026.1786849

**Published:** 2026-04-15

**Authors:** Shanza Rani, Muhammad Saleem Chang, Shuwei Li

**Affiliations:** 1 College of Life Sciences and Technology, Key Laboratory of Protection and Utilization of Biological Resources in Tarim Basin of Xinjiang Production and Construction Crops, Tarim University, Aral, China; 2 Department of Science & Technical Education, University of Sindh Jamshoro, Jamshoro, Sindh, Pakistan

**Keywords:** burn wound healing, flavonoid based, phytochemcial, quercetin, topical drug delivery

## Abstract

Burn injuries remain a significant global health challenge, often leading to prolonged healing, infection, and scarring. Addressing these complications requires innovative therapeutic strategies that can accelerate tissue repair and minimize adverse outcomes. Quercetin, a widely distributed plant-derived flavonoid, has emerged as a promising multi-functional agent in burn wound management. This review presents a comprehensive overview of quercetin’s therapeutic potential, emphasizing its pharmacological versatility and the need for advanced topical delivery systems such as nanostructured lipid carriers and hydrogels to overcome its limited bioavailability. Quercetin’s efficacy is rooted in its multi-targeted mechanisms of action. It exhibits potent antioxidant activity by directly scavenging reactive oxygen and nitrogen species and activating the Nrf2–ARE signaling pathway, thereby enhancing endogenous antioxidant defenses. Its anti-inflammatory effects are mediated through the inhibition of NF-κB and MAPK pathways, leading to the suppression of key pro-inflammatory cytokines. Quercetin also promotes angiogenesis via upregulation of vascular endothelial growth factor (VEGF), supports fibroblast proliferation and extracellular matrix remodeling, and facilitates re-epithelialization. This review consolidates dispersed experimental findings into a unified mechanistic and translational framework, highlighting why quercetin is uniquely positioned among phytochemicals for burn therapy. By integrating pharmacodynamics, formulation science, and clinical feasibility, the article clarifies current evidence gaps and outlines practical directions for therapeutic development. Such synthesis is timely because research on flavonoid-based wound therapeutics is expanding rapidly but remains fragmented across disciplines.

## 1 Introduction

Burn injuries remain a formidable medical challenge. Globally, the incidence is high, particularly in low- and middle-income countries, where resources for advanced wound care are limited (Irfan et al., 2022; Joshi et al., 2023). Beyond acute mortality, burns impose long-term burdens: secondary infections, prolonged hospitalization, scarring, contractures, and reconstructive surgeries. Effective therapies must do more than simply cover wounds-they must modulate the local microenvironment, suppress detrimental cascades, and promote regeneration (Chittasupho et al., 2002; Cetin et al., 2022; Kumar et al., 2025). Burn healing comprises overlapping phases: oxidative stress and inflammation, cell proliferation and angiogenesis, and remodeling of extracellular matrix (ECM). In thermal injury, the initial ROS/RNS burst damages cell membranes, nucleic acids, and proteins, amplifying tissue injury beyond the zone of direct thermal damage. A prolonged inflammatory milieu, with excess neutrophil infiltration and elevated cytokines (TNF-α, IL-1β, IL-6), further impedes transition toward repair. Meanwhile, microvascular damage restricts perfusion, and colonization by bacteria and biofilms threatens delayed healing or conversion to chronic wounds (Al-Shakarchi et al., 2022; Okselni A. et al., 2025; Monavari et al., 2024; Yadav et al., 2025; Badhwar et al., 2021).

The wound healing is one of the most critical processes of the physiology, which aims at restoring the materiality of the skin and the territory below it in a similar and simple content (Alizadeh and Ebrahimzadeh, 2022; Manginstar et al., 2024). The healing of wounds during burns and severe burns is especially significant to not only treat cosmetic problems but also precondition life-threatening problems (Chen et al., 2022; Huang et al., 2022). Burns can result in infections, over scarring and permanent disability in the absence of curing. One of the fundamental questions in the healing of burns is the maintenance of inflammatory response (Mi et al., 2022; Sierawska et al., 2022). Inflammation: This is a healing process because it is a natural process that cleans the wound and gets the tissue healing (Mulder et al., 2022; Orlińska et al., 2023). However, in case of chronic or excessive inflammation, it will result in the fact that the healing process will be slowed down, or the risk of infection and scarring will occur. Also, the burn injuries can destroy blood vessels that cause ineffective circulation in the damaged area and, therefore, complicate the healing of skin wounds (Qadir et al., 2021; Matar, et al., 2023). Therefore, burns treatment is a complex multi-phase procedure that involves inflammation, tissue regeneration, and skin remodeling (Ferreira et al., 2023; Lu et al., 2024). The healthy healing of wounds is an essential factor to prevent the complications such as infection, excessive scarring and prolonged pain (Orlińska et al., 2023; Skowrońska and Bazylko, 2023). Severe burns can overpower the body to the extent of the skin regenerating and healing being prolonged and taking extensive medical intervention (Zhao et al., 2021; Garg et al., 2025).

Thus, an ideal topical agent for burn therapy would combine antioxidant, anti-inflammatory, pro-angiogenic, ECM-regulating, and antimicrobial properties (Liu et al., 2022; Georgiou et al., 2023; Garg et al., 2024; Asgharnejad et al., 2025; Wu et al., 2025). Quercetin, a ubiquitous dietary flavonol, stands out in preclinical literature for its ability to engage multiple pathways simultaneously. Nrf2 pathway is very important in the regulation of antioxidant defenses like HO-1 and NQO1. Nrf2 signaling has been observed to be activated by quercetin. Moreover, recent mechanistic data has shown that quercetin-mediated stimulation of the Nrf2-ARE signaling axis is not only associated with antioxidant gene transcription but also coordinated regulation of phase II detoxifying enzymes, mitochondrial defence and inhibition of lipid peroxidation cascade, all of which enhances cytoprotective responses in tissue micro-environments of burn-damaged tissues. This could be accounted for by the long-term redox balance of the treated wounds due to this integrated pathway modulation. But its translation to clinical use has been hampered by poor solubility, photo lability, and limited penetration into target tissues. In recent years, advanced delivery technologies-such as hydrogels, nanostructured lipid carriers (NLCs), metal-organic frameworks (MOFs), core-shell nanofibers, and microneedles-have been developed to overcome these barriers. Several 2023–2025 studies including (Abdulrahman et al., 2025; Ahmed et al., 2024; Ali et al., 2017; Chen et al., 2022; Ding et al., 2023; Goyal et al., 2024) have demonstrated dramatic improvements in healing outcomes when quercetin is encapsulated into such systems (e.g., glycerohyalurosome hydrogel, quercetin bimetallic nanoparticles, textile nanofibers) (Sonawane and Pokharkar, 2024; Wang et al., 2022).

In this review, we present a detailed mechanistic synthesis, integrate the most recent quantitative preclinical evidence, compare quercetin to other phytochemicals, and chart actionable translational pathways-highlighting the key challenges, requisites of formulation and toxicology, and strategies for optimal clinical trial design.

### 1.1 Literature search strategy

The electronic databases, PubMed, Scopus, Web of Science, and Google Scholar were used to conduct a systematic search of literature to identify the required literature on this review. The search was conducted based on a combination of the following keywords quercetin, burn wound, skin injury, nanocarriers, hydrogels, and wound healing. Articles published since 2015 have been considered. The screening of the studies was done on basis of relevance, mechanistic focus, experimental design, and clarity of the methodology. Articles with no primary data, methodological transparency or criticality to burn-related mechanisms were filtered out. The graphical analyses were assembled with the help of Graphpad Prism and Python software to provide standard visualization.

## 2 Pharmacological profile and delivery challenges of quercetin

### 2.1 Chemical structure-activity: Relationships and pharmacodynamics

**FIGURE 1:**
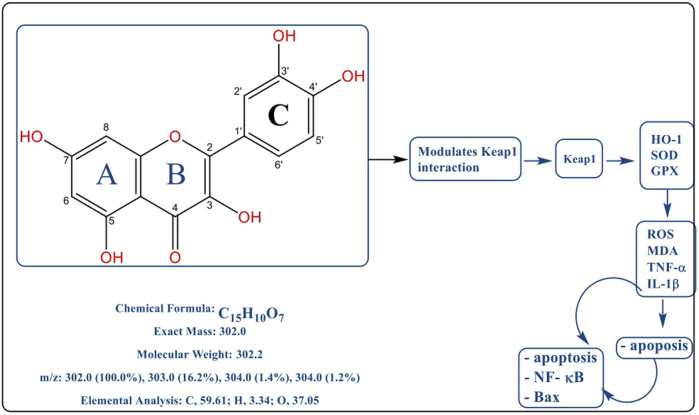
Schematic illustration of the reported pharmacodynamic mechanisms of quercetin in burn wound healing, based on previously published literature. The diagram summarizes how quercetin is described to influence the Keap1–Nrf2 pathway, antioxidant enzyme expression (HO-1, SOD, GPx), inflammatory mediators (TNF-α, IL-1β), and apoptosis-related signaling, as reported in earlier studies. (Note. All figures in this review are schematic illustrations created for explanatory purposes and are based exclusively on previously published literature).

Quercetin is a flavonol, a subclass of flavonoids, commonly found in plants in the form of O-glucosides. It possesses a characteristic three-ring structure with a C6-C3-C6 carbon skeleton, comprising two aromatic rings (A and B) and a heterocyclic pyrone ring (C ring), as illustrated schematically in Figure 1, based on previously published studies (Mirza et al., 2023). In addition, it contains 2, 3-double bonds and the five hydroxyl groups present at 3,5,7,3′,4′-positions with the 4-oxo group. Additional discussion of the structure-activity correlations indicate that the amount and location of hydroxyl replacements on the flavonol core greatly determine radical scavenging activity, metal chelation ability and permeability of a membrane. Specifically, the catechol structure in B ring and the 2,3-double bond conjugated with the 4-oxo group seem to be the ones that contribute to the electron delocalization, which is believed to be essential to stabilize the reactive oxygen species in oxidative stress-related to burning. Also, the 3-hydroxyl functional group can influence metal chelation (particularly of transition metal such as Fe 2 +/Fe 3 +, which suppresses Fenton-type reactions) and affinity binding of enzymes, with the planar conjugated structure also allowing p-p interactions and binding to a variety of protein targets (enzymes, receptors), which makes quercetin a so-called poly-pharmacological molecule (Bentz, 2017; Wu et al., 2025). Henceforth, a particular form can successfully ensure the quercetin can neutralize reactive oxygen species (ROS) effectively and serves to interact with a variety of proteins and molecular pathways in enzymes and signaling pathways, which underlies its pharmacological properties that affect healing processes in burn wounds in different directions (Tomou et al., 2023).

Quercetin’s glycosides (e.g., rutin, isoquercitrin) are more water-soluble, but once ingested, they undergo de-glycosylation and rapid phase II metabolism (glucuronidation, sulfation, methylation), yielding metabolites (e.g., quercetin-3′-O-glucuronide) with different bioactivities and kinetics. Thus, systemic delivery often results in low levels of unmetabolized quercetin at target sites, and the metabolites may not recapitulate the full spectrum of the parent compound’s pharmacology. Hence, topical delivery is preferred for localized action in burn wounds, as it bypasses first-pass metabolism and concentrates the active moiety at the site of injury (Chen et al., 2022; Singh et al., 2023; Terao, J. 2023; Mierczak and Garus-Pakowska, 2024; Čižmárová, et al., 2023).

**FIGURE 2:**
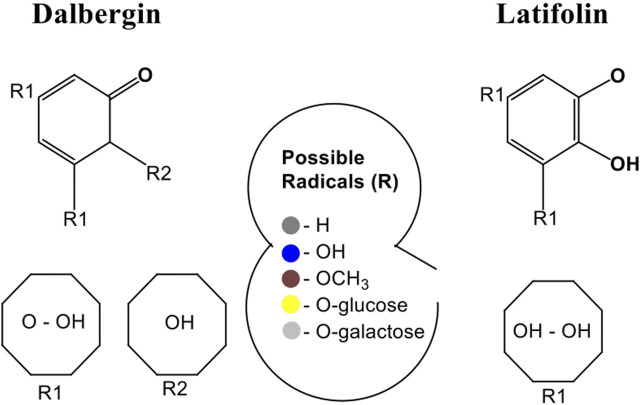
Literature-based schematic representation of Dalbergin and Latifolin derivatives showing core flavonoid scaffolds and representative substituent groups (R_1_, R_2_). The diagram was prepared using ChemDraw for illustrative purposes to support discussion of structure–activity relationships reported in previous studies.

On a similar vein, the pharmacological behavior of flavonoids is closely linked to their structural diversity, particularly the nature and position of substituent groups. The Dalbergin group (4-phenylcoumarins) and the Latifolin (diphenyl allyl compounds), two representative flavonoid scaffolds, exhibit variable biological activity depending on hydroxylation, methoxylation, and glycosylation patterns. Figure 2 presents a literature-based schematic of their derivatives, emphasizing how R-group substitutions modulate antioxidant and anti-apoptotic effects. However, these are not produced from edible plants but a variety of plants while depending on the nature of radicals attached, i.e., -OH, -OCH_3_, -glycoside, and -galactoside (Gomes et al., 2017). This structure–activity relationship (SAR) framework complements the pharmacodynamic analysis of quercetin and broadens the understanding of flavonoid-based therapeutic strategies.

### 2.2 Physicochemical barriers to tropical delivery

However, such a promising therapeutic application is completely impeded by its intrinsically subpar pharmacokinetic virtues (Mallya and Patil, 2021). Even so, topical delivery is not trivial. Quercetin has poor aqueous solubility (2.15 g/mL or 0.00215 g/L at 25 °C) due to the structure of its phenyl rings, which is a characteristic of aglycone form (Mallya and Patil, 2021). Likewise, it is moderately lipophilic (log P 1.8–2.5 at around 7.4 pH) that adds its crystalline form at body temperature, leads to low water solubility and is a highly susceptible to photochemical degradation and oxidation by ambient temperature and light (Mishra and Kulkarni, 2022; Carrillo-Martinez et al., 2024). Crespy et al. (1999) and Crespy et al. (2001) also attribute the poor bioavailability of quercetin aglycone and glycosides to their propensity (including that of their metabolites) to be effluxed back into the intestinal lumen after being taken up by the enterocytes. The intact stratum corneum represents a formidable barrier to diffusion; in burned or wounded skin, disrupted architecture, eschar, exudate, pH changes, and varied enzyme activity further complicate drug partitioning and stability. Lastly, Panthi et al. (2023) mentioned that there is a problem of local retention because of the turnover of wound fluids and mechanical loss (dressings displaced by movement, washing, or rubbing). Any effective topical clinical delivery must involve quercetin protection against degradation, increase in penetration into skin, controlled release to keep therapeutic concentrations, and retention (within a dressing matrix).

### 2.3 Advanced delivery strategies to overcome barriers

In recent years, researchers have developed a variety of innovative delivery systems for quercetin and related polyphenols to tackle major formulation challenges. These platforms aim to improve solubility, enhance stability, and boost skin absorption while ensuring targeted release (Shabir et al., 2022). Among them.

#### 2.3.1 Nanostructured lipid carriers (NLCs)

Lipid-based nanoparticles are generally divided into two categories: solid lipid nanoparticles (SLNs), which represent the first generation, and nanostructured lipid carriers (NLCs), the more advanced second generation (Komal and Singh, 2025). SLNs have fallen out of favor due to several drawbacks, including instability, limited drug loading efficiency, and a tendency toward polymorphic transitions. In contrast, NLCs offer a more versatile and effective alternative. When incorporated into hydrogel systems, they not only improve skin penetration and provide sustained drug release but also serve as a functional wound dressing. Their ability to deliver drugs in a controlled manner, along with ease of production and skin-moisturizing benefits, makes NLCs particularly promising for wound care applications (Chandana et al., 2019).

#### 2.3.2 Polysaccharide/composite hydrogels

Quercetin-loaded hydrogels have emerged as a promising solution for treating burn wounds. Their unique ability to maintain a moist environment while absorbing excess exudate, facilitating gas exchange, and delivering a cooling effect makes them particularly effective. Importantly, they ensure prolonged local delivery of quercetin without compromising therapeutic impact (Khatoon et al., 2022). These hydrogels are composed of three-dimensional networks of hydrophilic polymers that closely mimic the extracellular matrix, creating an ideal setting for tissue regeneration due to their high-water absorption capacity. In addition to maintaining optimal moisture levels, they help regulate temperature, stabilize pH, and alleviate pain during the healing process (Cai et al., 2024).

Natural polymers such as alginate, chitosan, hyaluronic acid, cellulose derivatives, and silk fibroin are commonly used to formulate hydrogels. These materials provide a moist wound environment, act as a barrier against external pathogens, and enable controlled drug release through diffusion or enzymatic degradation. A recent review by Ashfaq et al. (2025) highlights the benefits of quercetin-polysaccharide hydrogels, emphasizing their sustained antioxidant activity, ability to scavenge reactive oxygen species (ROS), and support for re-epithelialization. In a similar context, hydrogels infused with quercetin also demonstrate excellent physicochemical properties, including favorable swelling ratios, effective crosslinking, and optimal water vapor transmission rates. Studies involving bacterial cellulose and gelatin-based hydrogels have shown enhanced wound healing outcomes, including accelerated tissue repair, improved angiogenesis, and increased epithelial cell proliferation (Baharati and Lee, 2025).

#### 2.3.3 Metal-organic framework (MOF)/aerogel hybrid systems

One of the most advanced innovations in wound care is the ACA-MOF@Que aerogel, a therapeutic dressing that combines quercetin with amino-functionalized UIO-66 metal-organic frameworks (MOFs), all embedded within a polysaccharide-based aerogel. This smart system demonstrated remarkable healing potential in infected wound models, achieving nearly 95.5% closure by day 14. It also significantly boosted blood vessel formation and collagen buildup compared to untreated wounds. The study by Wu et al. (2025) highlighted the persistent hurdles in chronic wound healing, especially the need for sustained drug release and infection control. By encapsulating quercetin within MOFs, the formulation amplified its anti-inflammatory, antibacterial, and angiogenesis-promoting effects as key factors for tissue regeneration. The aerogel’s design leverages a pH-responsive release mechanism, allowing it to adapt to the wound’s microenvironment and optimize therapeutic impact. Lab and animal studies confirmed its ability to rapidly stop bleeding, reduce inflammation by guiding macrophages toward a healing-promoting state, and support tissue repair through enhanced vascularization and collagen synthesis (Wu et al., 2025).

Complementing this, the 2024 study introduced an innovative wound care approach using quercetin-loaded GelMA microneedles (Q-MNs), which showed impressive healing benefits. These microneedles create tiny channels that bypass the skin’s outer barrier, enabling quercetin to reach deeper layers of the dermis. This targeted delivery accelerated wound closure, boosted collagen production, and enhanced the formation of new blood vessels compared to conventional gels (Yi et al., 2024). The research team designed the Q-MN patch to improve healing outcomes, and lab tests confirmed its strong mechanical properties, excellent biocompatibility, and ability to support cell migration and tissue regeneration. In animal models with full-thickness skin wounds, Q-MNs significantly improved skin repair by promoting re-epithelialization and reducing oxidative stress. The patch also increased vascularization and collagen buildup, outperforming both untreated wounds and standard microneedle treatments. Key healing mechanisms included elevated levels of VEGF and reduced markers like MDA, indicating lower oxidative damage. Overall, the Q-MN system offers a promising and advanced solution for wound management, combining sustained drug release with enhanced healing, making it a strong candidate for future clinical applications.

#### 2.3.4 Core–shell electrospun nanofibers and nanoparticle-based modifications

In the field of burn wound care, Monavari et al. (2024) introduced a novel nanofiber dressing designed to deliver both antimicrobial and healing support. This advanced material features a core-shell structure, where the inner layer contains the antibiotic levofloxacin (LEV) and the outer shell is infused with quercetin (QS), a compound known for its antioxidant and regenerative properties. By combining these two agents, the dressing offers a dual-action approach, as fighting infection while promoting tissue repair. Laboratory and animal studies confirmed its effectiveness, showing faster wound closure and improved healing outcomes. These benefits are likely tied to the dressing’s ability to reduce bacterial presence and oxidative stress at the injury site, creating a more favorable environment for recovery.

On a similar vein, quercetin-functionalized, green-synthesized iron and zinc oxide bimetallic nanoparticles (ZFQNP) offered enhanced wound-healing properties compared to free quercetin, likely due to improved delivery, protection, and perhaps catalytic interactions at the wound milieu (Yadav et al., 2025). The study demonstrated that these bimetallic nanoparticles could serve as a promising therapeutic platform for skin repair. By leveraging the synergistic effects of iron and zinc oxides, the formulation not only stabilized quercetin but also amplified its biological activity. *In vivo* experiments using excisional wound models in rats revealed that ZFQNP-based ointments significantly accelerated wound closure, likely through modulation of key healing pathways. Molecular docking studies further supported the strong binding affinity of quercetin to wound-related protein targets, suggesting a mechanistic basis for its enhanced efficacy. Overall, the integration of quercetin into a bimetallic nanoparticle system presents a compelling strategy for promoting tissue regeneration, reducing inflammation, and improving the overall healing trajectory.

Moreover, Yang et al. (2023) developed a gallium-modified gelatin nanoparticle system infused with quercetin that demonstrated multifaceted benefits in skin wound healing. Beyond accelerating tissue repair, the formulation exhibited strong antibacterial activity and helped minimize scar formation. These effects were largely attributed to its ability to influence immune cell behavior-specifically, by shifting macrophages from a pro-inflammatory M1 state to a healing-promoting M2 phenotype. This transition was mediated through activation of the TGF-β/Smad signaling pathway, which plays a key role in regulating inflammation and tissue regeneration. The study highlights the therapeutic potential of combining gallium’s antimicrobial properties with quercetin’s anti-inflammatory and regenerative effects in a nanoparticle-based delivery system.

#### 2.3.5 Silk fibroin/soybean protein isolate (SF/SPI) hydrogels with quercetin

**FIGURE 3:**
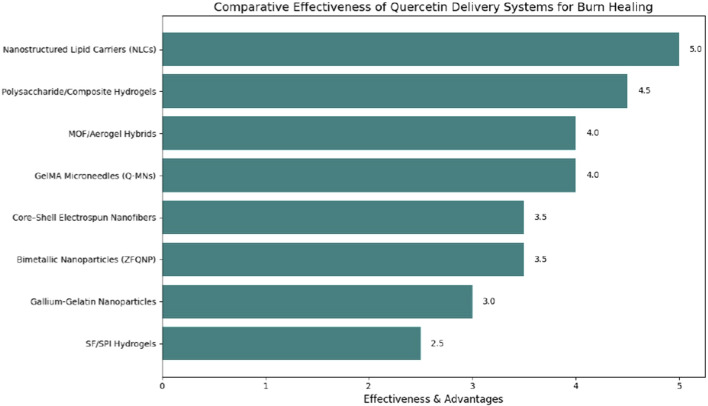
Comparative efficacy of quercetin delivery systems. The vertical axis represents percentage wound closure, while the horizontal axis indicates treatment duration in days. Values are derived from reported experimental datasets.

Recent advancements in wound care have focused on combining bioactive compounds with smart delivery systems to accelerate healing and reduce complications. One such approach was demonstrated by Li et al. (2024), who developed silk fibroin/soybean protein isolate hydrogels loaded with quercetin (SF/SPI-Q). These hydrogels significantly enhanced epidermal regeneration, collagen deposition, and angiogenesis in burn wound models. The study highlighted how blending natural proteins with therapeutic agents can create multifunctional dressings that support tissue repair while maintaining biocompatibility and biodegradability. This aligns with other emerging strategies, such as the gallium-loaded gelatin nanoparticles carrying quercetin developed by Yang et al. (2023), and quercetin-functionalized iron and zinc oxide bimetallic nanoparticles (ZFQNP), which outperformed free quercetin in wound healing efficacy introduced by Yadav et al. (2025). Together, these studies underscore a growing trend in wound therapy, integrating natural compounds like quercetin with engineered carriers to create responsive, multifunctional platforms that address infection, inflammation, and tissue regeneration simultaneously. These technologies illustrate that the primary barrier to quercetin’s translational use is not a lack of biological efficacy, but the engineering challenge of delivery (Figure 3).

Though various platforms of delivery have been researched, the comparative functioning of delivery platforms differs significantly. Based on penetration between the stratum corneum, lipid-based carriers prove to be more effective, but they might experience stability problems when stored. Polymeric nanoparticles are controlled release but they are limited in drug loading capacity in certain instances. Hydrogels offer a moist healing condition and prolonged exposure to the drug, but the mechanical strength may not be adequate to large burns. There is no single formulation that is universally the best and the selection of the system must be informed by the severity of the wound, the release kinetics that are required and clinical practicability as opposed to the novelty of the formulation.

### 2.4 Comparative evaluation of delivery platforms

Even though numerous delivery systems have been proven to enhance better therapeutic performance, their benefits vary according to formulation properties. Lipid nanoparticles have better penetration and controlled release, but they can have a stability problem during storage. The NLCs therefore have a better prospective to other LNP formulations like high drug loading capacity, controlled release and enhanced stability in the drug delivery (Lawson, 2023). There is a potential of synergy between quercetin-loaded hydrogel and metal-organizational frameworks (MOFs) to address the shortcomings of each constituent. Hydrogels, though offering moisture retention and localized drug action to wound healing, do not have the mechanical strength necessary to be stable. On the other hand, MOFs have high drug loading capacity and release profiles but pose a risk in terms of biocompatibility and biodegradation in the long run (Kush et al., 2025). Quercetin-loaded electrical sprayed nanofibers are designed to follow the pathologic framework of the native extracellular matrix (ECM), which offers highly permeable, 3D scaffolds that encourage cell adhesion, cell growth, and cell migration. The presence of quercetin in these nanofibrous mats (in most cases with polymers such as PCL, gelatin, or PLA) boosts biological functions such as antioxidant effects, wound healing, and drug delivery (Karuppannan et al., 2022). Therefore, there is no universal superiority of one system over the other and the choice of the platform must be determined by the targeted clinical goal, wound severity, and release kinetics needs.

## 3 Mechanism of action in burn healing

The remarkable wound-healing potential of quercetin in burn injuries, as demonstrated in numerous preclinical studies, cannot be attributed to a single mechanism.

**FIGURE 4:**
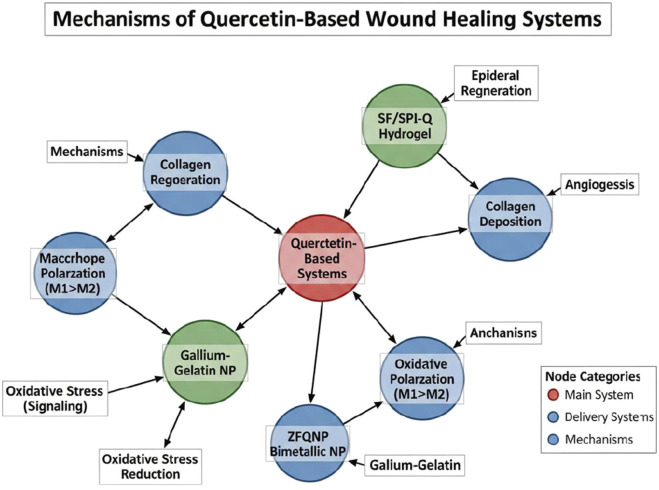
Mechanism of Quercetin-based wound healing system.

Rather, it stems from its multi-targeted nature, modulating a complex network of overlapping biological processes essential for tissue repair (Mi et al., 2022). As Xing et al. (2024) emphasized, quercetin’s therapeutic effects are orchestrated through a cascade of tightly regulated molecular events that respond to the pathophysiological challenges posed by burn trauma, an overview of which is illustrated in Figure 4.

One of quercetin’s most immediate and powerful actions is its regulation of oxidative stress (Kartal et al., 2024). Thermal injury triggers a surge in reactive oxygen and nitrogen species (ROS and RNS), which aggressively oxidize cellular lipids, proteins, and DNA, exacerbating tissue damage and impairing healing. Quercetin acts as a potent first-line antioxidant, donating electrons to neutralize these free radicals before they inflict cellular harm (Panthi et al., 2023). Beyond this direct scavenging role, quercetin activates the nuclear factor erythroid 2-related factor 2 (Nrf2) signaling pathway, a key regulator of intrinsic antioxidant defenses. Under oxidative stress, quercetin facilitates Nrf2 translocation into the nucleus, where it binds to the Antioxidant Response Element (ARE) and promotes transcription of cytoprotective enzymes such as superoxide dismutase (SOD), catalase (CAT), and glutathione peroxidase (GPx), thereby restoring redox balance and supporting tissue recovery (Priyadarshi et al., 2022; Widiatmoko et al., 2024). Researchers discovered that quercetin reversed the cumulative expression of NADPH oxidase in oxLDL and the oxLDL-stimulated declines of activation of AMP-activated protein kinase, thus preventing NF-kB signals and holding AKT/eNOS activity (Hung et al., 2015). Moreover, Xu et al. (2019) supported this observation and showed that quercetin enhances the antioxidant properties of cells by promoting the activation of intracellular p38 MAPK pathway, increasing intracellular GSH levels, and serving as a source of hydrogen donors during free radical reactions scavenging. It is known that bad environmental conditions enhance ROS production. The factors promote the effect of the electron transport chains of mitochondria, which make a significant source of intracellular production of ROS (Xu et al., 2019). The body combats the free radicals with the help of two primary defense mechanisms: non-enzymatic antioxidants consisting of vitamins and trace elements (vitamin C, vitamin E, selenium, copper, manganese, etc.) and enzymatic antioxidants consisting of SOD, including catalase, glutathionase, etc. Quercetin can stimulate the antioxidant defense system and ensure the oxidative homeostasis not only by balancing the non-enzymatic-dependent antioxidant defense system.

Simultaneously, quercetin exerts potent anti-inflammatory effects, counteracting the excessive inflammatory response typical of burn wounds. It suppresses key pro-inflammatory signaling pathways, notably nuclear factor-kappa B (NF-κB) and mitogen-activated protein kinase (MAPK) (Hajam et al., 2025). By inhibiting IκB degradation and preventing NF-κB nuclear translocation, quercetin downregulates the expression of inflammatory mediators such as tumor necrosis factor-alpha (TNF-α), interleukin-1 beta (IL-1β), and interleukin-6 (IL-6), which are otherwise elevated and contribute to pain and tissue destruction. Additionally, quercetin modulates immune cell behavior, reducing neutrophil infiltration and promoting macrophage polarization from the pro-inflammatory M1 phenotype to the pro-healing M2 phenotype thus facilitating the resolution of inflammation and transition to the proliferative phase (El-Sherbeni and Negm, 2023; Hajam et al., 2025).

Following the acute inflammatory stage, angiogenesis becomes critical for delivering oxygen and nutrients to regenerating tissue. Quercetin enhances this process by upregulating vascular endothelial growth factor (VEGF), the master regulator of angiogenesis, and amplifying VEGF-mediated signaling (Kumar and Sharma, 2023). This stimulates endothelial cell proliferation and migration, leading to the formation of new capillary networks that restore perfusion to the ischemic wound bed and support ongoing tissue repair (Zhu et al., 2024).

During the proliferative phase, quercetin activates fibroblasts and promotes their differentiation into myofibroblasts via the transforming growth factor-beta (TGF-β1) pathway (Arghand et al., 2024). This leads to robust synthesis and deposition of extracellular matrix (ECM) components, particularly collagen types I and III and fibronectin, which are essential for granulation tissue formation. Notably, quercetin enhances collagen fiber organization, cross-linking, and maturation, thereby increasing the tensile strength of newly formed tissue and reducing the risk of abnormal scarring (Alharbi et al., 2025).

Infection remains one of the most serious complications in burn wounds, and quercetin offers direct antimicrobial and antibiofilm activity. It exhibits bactericidal effects against common burn pathogens such as *Staphylococcus aureus* and *Pseudomonas aeruginosa* by disrupting bacterial cell walls and interfering with nucleic acid synthesis (Du et al., 2025). More importantly, quercetin impairs biofilm formation and destabilizes existing biofilms, structured microbial communities that are notoriously resistant to antibiotics. It enhances the efficacy of conventional antimicrobials by modulating quorum sensing and downregulating biofilm-associated genes, thereby forming a protective barrier against infection (Shubina et al., 2024; Li et al., 2024). A summary of these mechanisms is presented in Table 1.

**TABLE 1:** Summary of quercetin’s biological effects and mechanisms in burn wound healing. Each category reflects a key therapeutic action supported by molecular and cellular pathways described in recent literature.

Mechanism of action	Key molecular targets/Pathways	Biological effect & outcome
Antioxidant	Direct ROS/RNS scavenging; activation of Nrf2-ARE pathway	Reduces oxidative damage to cells; upregulates antioxidant enzymes (SOD, CAT, GPx); creates a redox-balanced microenvironment conducive to healing
Anti-inflammatory	Inhibition of NF-κB and MAPK signaling pathways	Downregulates pro-inflammatory cytokines (TNF-α, IL-1β, IL-6); reduces neutrophil infiltration; promotes macrophage polarization to pro-healing M2 phenotype
Pro-angiogenic	Upregulation of vascular endothelial growth factor (VEGF)	Stimulates the formation of new blood vessels (angiogenesis); improves oxygen and nutrient supply to the wound bed; supports granulation tissue formation
Tissue regeneration	Enhanced TGF-β1 signaling; fibroblast proliferation	Stimulates synthesis and deposition of collagen types I and III and fibronectin; improves collagen fiber organization and tensile strength; reduces risk of abnormal scarring
Re-epithelialization	Promotion of keratinocyte migration and proliferation; inhibition of apoptosis	Accelerates the regeneration of the epidermal layer; quickly restores the skin’s protective barrier
Antimicrobial & antibiofilm	Disruption of bacterial cell walls; inhibition of nucleic acid synthesis; interference with quorum sensing	Direct activity against common burn pathogens (e.g., *S. aureus*, *P. aeruginosa*); disrupts and prevents biofilm formation; can synergize with conventional antibiotics

Finally, quercetin plays a pivotal role in re-epithelialization, the final stage of wound healing. These results were further supported by quantitative measurement of epithelial thickness, collagen fibers orientation and fibroblast density, which was an indication of not only accelerated closure but also better architecture of tissues. This type of structural organization is typically linked to the decreased amount of scarring and the increased biomechanical ability of regenerated skin (Wu et al., 2025). It protects fibroblasts and keratinocytes from apoptosis induced by oxidative stress and inflammatory cytokines, preserving the cellular machinery required for tissue regeneration (Jafar and Shami, 2025). Moreover, quercetin promotes keratinocyte proliferation and migration at the wound margins, accelerating epidermal coverage and restoring the skin’s protective barrier. Through this coordinated suite of actions like antioxidant, anti-inflammatory, pro-angiogenic, fibroblast-stimulating, antimicrobial, and epithelial-regenerating, quercetin addresses the multifaceted challenges of burn wound healing and offers a comprehensive therapeutic approach (Falbo et al., 2023).

## 4 Preclinical evidence supporting quercetin in burn wound healing

The rationale for using quercetin in burn wound therapy is increasingly supported by a growing body of *in vivo* research, particularly in rodent models (Huang et al., 2024). These studies consistently demonstrate that quercetin-based formulations, especially when applied to second-degree partial-thickness burns, outperform both untreated and conventionally treated controls. Across multiple investigations, a recurring pattern of therapeutic benefit emerges, reinforcing quercetin’s potential as a wound-healing agent (Irfan et al., 2022).

One of the earliest studies to explore quercetin’s topical application used a simple cream formulation (0.3% w/w), administered twice daily. In a rat model of second-degree burns, this treatment significantly accelerated wound closure compared to the vehicle control (Zulkefli et al., 2023). Histological analysis revealed reduced inflammatory cell infiltration, enhanced granulation tissue formation, and improved collagen fiber organization. These findings suggest that even without advanced delivery systems, quercetin can positively modulate the wound microenvironment to support healing (Nisar et al., 2023). Also, time-specific analysis showed that initial inflammatory-modulating effect, as indicated by the presence of visible macroscopic wound healing, was preceded by quercetin activities modulating the biochemical wound microenvironment, thereby implying that quercetin would be an agent that primarily influences the structural repair before its manifestation. This chronological difference reinforced the mechanistic viability of its treatment effect (Yadav et al., 2025).

**FIGURE 5:**
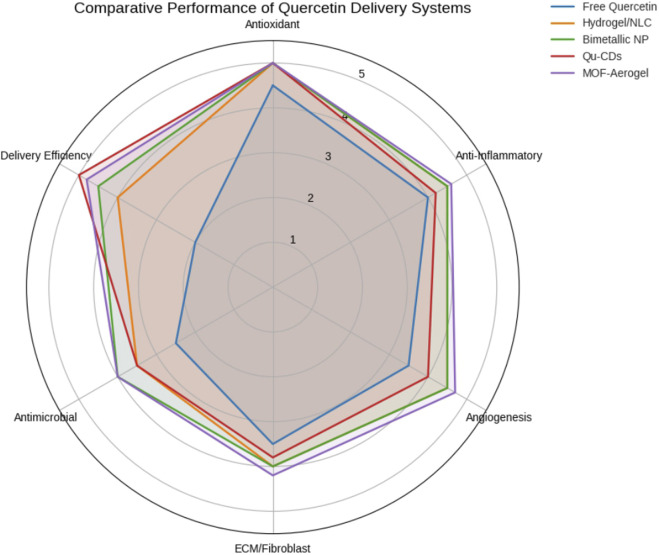
Radar chart comparing the normalized performance of five quercetin delivery systems in burn wound models, based on data extracted from previously published studies. Scores reflect relative efficacy across antioxidant, anti-inflammatory, angiogenic, regenerative, antimicrobial, and delivery efficiency parameters.

Recognizing the limitations of basic formulations, more recent studies have focused on enhancing quercetin’s bioavailability through nanocarrier systems. Among these, nanostructured lipid carriers (NLCs) have shown promise. In a comparative study involving second-degree burns, quercetin-loaded NLCs (0.3% w/w) were evaluated against free quercetin and silver sulfadiazine (SSD) cream. The NLC group exhibited superior healing outcomes, including reduced inflammation, increased fibroblast activity, thicker and better-aligned collagen layers, and earlier re-epithelialization (Chaturvedi et al., 2023; Faour, 2023). These results underscore the value of advanced nano formulations in unlocking quercetin’s full therapeutic potential. Probably, the interconnected molecular pathways, affected by quercetin, have been summarized as Figure 5. The illustration shows that pro-inflammatory cytokines are suppressed, antioxidant enzyme activated, fibroblast proliferation stimulated, and collagen deposition controlled. All these pathways describe the way that one compound can affect various phases of wound repair.

While topical application remains the preferred route, systemic effects have also been explored. In one study, a quercetin-phospholipid complex (phytosome) was administered orally to burned rats. The treatment reduced wound size, improved histological features, and enhanced collagen deposition while reducing edema (Suda et al., 2025). Importantly, systemic oxidative stress markers were normalized, with increased serum levels of SOD and GPx and decreased MDA, suggesting that quercetin’s benefits may extend beyond the local wound site.

Taken together, these findings offer compelling preclinical evidence for quercetin’s broad-spectrum efficacy across different rodent strains and burn models (Gál et al., 2023). Consistent outcomes include faster wound closure, reduced wound area, attenuated inflammation, enhanced collagen synthesis and organization, stimulated angiogenesis, and accelerated re-epithelialization. Elevated antioxidant levels in wound tissue further support its therapeutic profile (Kowalski et al., 2024). Notably, advanced formulations, particularly NLCs and hydrogels have consistently matched or exceeded the performance of SSD, with NLCs showing superior tissue regeneration and added antimicrobial benefits (Gahtori et al., 2023). These findings provide a strong foundation for advancing quercetin toward clinical application in burn care. Although the preclinical rodent burn models would offer useful mechanistic understanding, they must be translated to the human clinical environment with careful consideration of interspecies differences in skin thickness, immune response, metabolic rate, and wound contraction patterns. Clinical trials with controlled conditions, dose regimens and long-term safety follow-ups will thus be necessary before routine therapeutic implementation can be warranted. A comparative synopsis of preclinical investigations on quercetin-based wound dressing treatments of burn wounds is presented in Table 2.

**TABLE 2:** Comparative summary of preclinical studies evaluating quercetin-based burn wound therapies.

Study	Model	Formulation	Key outcomes	Limitations	Further trials needed
Kant et al. (2021)	Rat burn	0.3% ointment	Faster closure, repair and regeneration of cutaneous diabetic wounds	Small sample	Yes
Xing et al. (2024)	SD rats (deep 2nd-degree)	Quercetin-loaded polysaccharide (PECE) injectable hydrogel	Scarless healing; reduced IL-6/TNF-α; enhanced angiogenesis and collagen deposition	Complex fabrication process; focus on deep 2nd degree only	Yes
Cetin et al. (2022)	Wistar albino rats	Quercetin-loaded PLGA nanoparticles and thermosensitive gel	Accelerated wound closure; gel formulation was more cost-effective than nanoparticles	Low encapsulation efficiency (25%) for nanoparticles	Yes (optimizing nano-dosage)
Karuppannan et al. (2022)	Wistar rats	Quercetin-loaded PCL/Gelatin electrospun nanofibers	Complete wound contraction in 18 days; sustained drug release; high mechanical strength	*In vitro* heavy; short *in vivo* monitoring period	Yes (chronic burn models)
Chittasupho et al. (2022)	Human fibroblasts (in vitro)	Quercetin + curcuminoid (3:1) mixture	Synergistic effect: Higher antimicrobial activity against *P. aeruginosa* and *S. aureus*	Lacks full *in vivo* burn model validation	Yes (in vivo animal studies)
Irfan et al. (2022)	Wistar rats (cold burn)	Quercetin-preconditioned mesenchymal stem cells (hUC-MSCs)	Remarkable remodeling at day 14; upregulation of VEGF/FGF; reduced oxidative stress	Complexity of combining stem cells with flavonoids	Yes (clinical safety trials)
Jangde et al. (2018)	Albino rats	Quercetin-loaded liposomal multiphase hydrogel	Significant decrease in closure time compared to conventional cream; high stability	Limited comparison with modern nano-dressings	Yes (comparison with SSD)
Doersch et al. (2017)	*In Vitro* & rat models	Quercetin solution (topical)	Reduced fibrosis via modulation of αV and β1 integrin expression on fibroblasts	Rapid degradation of quercetin in solution form	Yes (sustained-release forms)
Bhatia et al., 2016	Rats (scald burn)	0.06% isoquercetin-based cream	Epithelialization in 10.8 days (faster than silver sulfadiazine at 15.6 days)	Focus on isoquercetin (derivative) rather than pure quercetin	Yes (dose-response optimization)

## 5 Potential for clinical translation and current challenges

### 5.1 From bench to bedside: A multi-targeted therapeutic rationale

Quercetin’s progression from a promising preclinical compound to a viable therapeutic candidate for burn wound healing is underpinned by a robust and well-articulated scientific foundation (Panthi et al., 2023). As outlined in earlier sections, quercetin is far more than a symptomatic agent-it acts as a comprehensive modulator of the wound healing cascade. Its mechanism of action is notably holistic, targeting multiple interconnected pathological processes triggered by burn injuries.

At the molecular level, quercetin neutralizes reactive oxygen and nitrogen species, thereby limiting oxidative damage. Furthermore, Nrf2/Keap1 pathway is critical in ensuring that cells are resistant to oxidative debilitation by controlling the expression of numerous antioxidant proteins. Quercetin enhances gene expression of antioxidant response involving Nrf2, leading to the reduction of oxidative stress and the damage of cells. Such modulation is essential to mitigate the harmful consequences of reperfusion injury since it can be used to sustain cellular homeostasis and avoid a high inflammatory state (Yousefi Zardak et al., 2024). In particular, the downregulation of pro-inflammatory mediators including TNF-a, IL-1b, and IL-6 indicate that quercetin could act as an interference with NF-kB signaling, which is a pathway that is commonly involved in the chronic wound inflammation. Past research has reported crosstalk between Nrf2 and NF-kB signaling and the interaction may be the explanation of the dual antioxidant and anti-inflammatory effects observed in the current study (Yousefi Zardak et al., 2024).

This multi-targeted pharmacological profile stands in contrast to conventional treatments like silver sulfadiazine, which primarily offer antimicrobial effects and may even delay healing due to cytotoxicity (Alberts et al., 2025). The potential of a single compound to address oxidative stress, inflammation, infection, and tissue repair is compelling. It opens the door to simplified treatment regimens that could improve patient compliance, reduce healthcare costs, and accelerate recovery with minimal scarring (Molaei et al., 2025). The consistent success of quercetin across various animal models and advanced formulations provides a strong foundation for initiating clinical trials. Many researchers now consider quercetin a biologically potent candidate with the potential to redefine burn care (Sood et al., 2025 a clinically approved therapy presents several significant challenges (Utpal et al., 2025). One of the foremost obstacles is the development of effective topical delivery systems. Quercetin’s lipophilic nature and poor water solubility limit its ability to reach therapeutic concentrations in the wound bed, especially in necrotic or eschar-covered tissue (Bhati et al., 2024). Simply incorporating raw quercetin into a cream or ointment is unlikely to replicate the results seen in controlled animal studies.

Therefore, successful clinical translation hinges on the advancement of specialized drug delivery technologies (Bhuyan et al., 2024). Nanostructured lipid carriers (NLCs), hydrogels, and liposomes have emerged as promising platforms, not merely as carriers but as therapeutic enhancers. These systems stabilize quercetin, improve its solubility, enable sustained release, and enhance tissue penetration (Rattanachot et al., 2025; Xiong et al., 2024). The choice of delivery system must also be tailored to the wound’s characteristics-hydrogels may be ideal for moist, exudative wounds, while occlusive formulations could be more suitable for dry or healing stages. Ultimately, the clinical question is not just whether quercetin works, but whether a specific, optimized, and scalable formulation can deliver consistent therapeutic outcomes (Chen et al., 2022).

Closely tied to delivery is the issue of dosing and standardization. Preclinical studies have used a wide range of concentrations (typically 0.3%–1% w/w), but no optimal human dose has been established. Phase I clinical trials are essential to assess safety and tolerability, followed by Phase II studies to determine the most effective dosing regimen-including concentration, frequency, and duration of application (Guan et al., 2025). Standardization is particularly critical given quercetin’s natural origin. Its chemical profile and potency can vary depending on botanical source and extraction method. To qualify as a pharmaceutical agent rather than a dietary supplement, manufacturers must ensure high-purity, batch-consistent formulations with rigorous quality control (Scattolin et al., 2024).

Perhaps the most significant gap in the current evidence base is the absence of human clinical trials. While animal models are invaluable for mechanistic insights, they do not fully replicate the complexity of human burn wounds, which may be influenced by factors such as age, diabetes, or malnutrition (Li et al., 2025). Randomized controlled trials (RCTs) remain the gold standard for evaluating efficacy in humans. There is an urgent need for well-designed, double-blind, placebo-controlled RCTs comparing optimized quercetin formulations to both placebo and standard treatments like silver sulfadiazine or modern silver-based dressings (Raheem et al., 2025).

Primary endpoints should include wound closure rate, healing time, and infection incidence, while secondary outcomes might assess pain levels, scar quality (e.g., via the Vancouver Scar Scale), and patient-reported satisfaction. Safety must also be rigorously evaluated. Although quercetin is Generally Recognized as Safe (GRAS) for dietary use, topical application on compromised skin presents different risks (Zaborowski et al., 2024). Damaged skin may allow systemic absorption, necessitating toxicological studies to rule out local irritation, allergic reactions, and systemic toxicity. Monitoring renal and hepatic function will be essential to ensure that quercetin and its metabolites do not accumulate to harmful levels (Lee and Kim, 2022).

In summary, while quercetin holds significant promise as a next-generation therapy for burn wound healing, its clinical adoption depends on a careful, stepwise approach. This includes formulation refinement, dose optimization, product standardization, and comprehensive human safety and efficacy trials (Shivgotra et al., 2025).

## 6 Comparative analysis with other natural compounds in burn healing

The use of phytochemicals in burn wound therapy is a rapidly expanding area of research, with numerous natural compounds demonstrating therapeutic potential. Within this landscape, quercetin stands out for its broad-spectrum efficacy and well-characterized mechanisms of action, making it a strong candidate for both standalone and combination therapies. Thus, the most considerable concentrations of quercetin are found in many plant foods such as capers, rocket, dill, coriander, fennel, juniper berries, corn poppy, bee pollen and okra (Suganthy et al., 2016). The given variegated provenances of quercetin show that there are country-specific and regional deviations that may be explained by the complexity of local conditions, traditions, and other aspects (Yi et al., 2021). Besides that, quercetin is found in over twenty plant species such as *Santalum album*, *Mangifera indica*, *Emblica ofcinalis*, *Curcuma domestica valenton*, *Withania somnifera*, *Foeniculum vulgare*, and *Cuscuta refexa*. It contains different levels of concentration varying with different species, conditions of cultivation and processing methods. An extraction of plant matrices is normally followed by solvent fractionation or chromatographic separation in processing industrial production. Alternative synthetic and semi-synthetic methods have been tried, such as the use of chalcone intermediates in constructing a flavonol ring. The process of natural extraction is less damaging to the environment, yet the amount of it can be smaller, and the process of synthetic production, though more costly and with increased amounts of chemicals released into the environment, is less damaging. Production method choice thus remains in the hands of the desired use of the pharmaceutical and the bottom line.

Several natural agents have shown effectiveness in specific aspects of burn healing, each with distinct pharmacological profiles. For example, curcumin, the active compound in turmeric, exhibits potent antioxidant and anti-inflammatory properties. It suppresses NF-κB signaling and downregulates pro-inflammatory cytokines, similar to quercetin. However, curcumin’s poor chemical stability and limited bioavailability pose significant challenges, requiring advanced delivery systems to achieve therapeutic relevance (Oskelni T. et al., 2025; Xu et al., 2025).

Aloe vera, long used in traditional medicine, offers cooling and moisturizing effects for superficial burns. Its polysaccharide component, acemannan, stimulates macrophage activation and fibroblast proliferation. Yet, its efficacy diminishes in deeper burns, where regenerative demands are higher (Xu et al., 2025). Medical-grade Manuka honey is widely recognized for its antimicrobial activity, ability to maintain a moist wound environment, and moderate anti-inflammatory effects. Its high osmolarity and hydrogen peroxide production contribute to wound cleansing and bacterial inhibition. However, its action is largely physical and microbiological, lacking the molecular signaling modulation seen with quercetin (Huang et al., 2024).

Chitosan, a biopolymer derived from chitin, is frequently used as a scaffold or hydrogel base in drug delivery systems. It possesses intrinsic hemostatic, antimicrobial, and wound-healing properties, primarily through electrostatic interactions with microbial membranes and red blood cells. Its therapeutic potential is significantly enhanced when combined with bioactive agents like quercetin, forming synergistic systems that offer both structural support and targeted pharmacological action (Oskelni T. et al., 2025).

Other notable phytochemicals include silymarin, a flavonoid complex from milk thistle, and resveratrol, a stilbene found in grapes. Both exhibit strong antioxidant and anti-fibrotic effects, which may contribute to burn repair. However, compared to these compounds, quercetin’s comprehensive mechanistic profile is unmatched. It is one of the few natural agents with robust preclinical evidence demonstrating simultaneous modulation of oxidative stress, inflammation, angiogenesis, fibroblast activation, collagen synthesis, re-epithelialization, and antimicrobial activity (Oskelni T. et al., 2025; Xu et al., 2025).

This multi-targeted efficacy is further supported by biomarker-level evidence from recent meta-analyses and preclinical studies, summarized in Table 3.

**TABLE 3:** Representative biomarker changes associated with quercetin treatment in burn wound models.

Marker	Direction of change	Representative range (% vs. control)	Biological interpretation	Key sources
MDA	↓	40%–65%	Reduced lipid peroxidation	Oskelni A. et al. (2025); Xu et al. (2025)
SOD	↑	40%–80%	Enhanced superoxide dismutase activity	Oskelni A. et al. (2025); Xu et al. (2025)
CAT	↑	30%–60%	Catalase induction	Oskelni A. et al. (2025); Xu et al. (2025)
GSH	↑	50%–100%	Replenished glutathione pool	Oskelni A. et al. (2025); Xu et al. (2025)
IL-1β, TNF-α	↓	40%–70%	Reduced pro-inflammatory cytokines	Huang et al. (2024)
VEGF	↑	40%–90%	Pro-angiogenic response	Xu et al. (2025)
Hydroxyproline	↑	60%–100%	Collagen synthesis	Oskelni A. et al. (2025)
α-SMA	↑	50%–70%	Myofibroblast differentiation	Oskelni A. et al. (2025)

These quantitative findings reinforce quercetin’s superiority in modulating key biological processes involved in burn healing. Its ability to simultaneously reduce oxidative stress and inflammation, while promoting angiogenesis and tissue regeneration, makes it especially attractive as a core component of advanced wound therapies. In comparison to the previous flavonoid-based burn treatment literature, the extent of healing speed here is in line with reports that show that plant-based polyphenols have the potential to stimulate the process of repairing the skin with respect to collagen production and angiogenesis. This regularity adds to the extravasation validity of the current results. Notably, the identified enhancement of the healing parameters seems to be multifactorial as opposed to a biochemical process. Quercetin also balances the oxidative stress, inflammatory pathways and proliferation of cells. This multitask activity is the unique feature of it in comparison with the one-target pharmacologic agents and can explain its increased efficacy in complex tissue-injury models, including burns. Dose optimization is another important factor. The polyphenolic compounds have frequently been found to have biphasic dose-response relationships with low to moderate dose having protective effect and excessive high dose having pro-oxidant action. More research in the future must then involve dose-range discovery as appropriate therapeutic windows. Future research should focus on direct comparative studies using optimized formulations, such as quercetin-loaded NLCs or hydrogels against other high-grade natural compounds to establish a hierarchy of therapeutic potential. Additionally, rational combination therapies offer exciting possibilities. For instance, a quercetin-curcumin nano-formulation could amplify anti-inflammatory effects, while a quercetin-infused Manuka honey hydrogel might synergize antimicrobial and regenerative properties. These integrative approaches could redefine natural product-based wound care by combining molecular precision with broad-spectrum healing. Although the results are promising, there should be several limitations. To begin with, the preclinical models used as experimental designs might not necessarily mimic the human wound physiology. Second, pharmacokinetic parameters (absorption, distribution, metabolism and excretion) were not directly measured. Third, the study was not long enough to determine the quality of the scar in the long term. These limitations are necessary to be addressed in future work to warrant the relevance of translations.

Clinically, quercetin has the potential to be investigated as a topical adjunctive agent in the management of burns especially in the environment where low-cost treatments are required. The natural origin of it has defined safety profile in dietary exposure, and has wide-ranged pharmacological activity; thus, it is a promising candidate in further pharmaceutical formulation development, such as hydrogel, nano emulsions, and polymeric wound covers that are aimed at promoting skin penetration and prolonged release.

In conclusion, while many phytochemicals contribute meaningfully to burn healing, quercetin’s depth of evidence and multi-dimensional activity make it one of the most compelling candidates for further development. It’s potential to serve as both a therapeutic agent and a delivery system enhancer warrants serious consideration in future drug development strategies.

## 7 Conclusion and future perspectives

This comprehensive review presents a compelling and multidimensional case for the therapeutic application of quercetin in burn wound management. The most consistent and impactful findings across mechanistic and preclinical studies highlight quercetin’s ability to regulate the entire wound healing cascade, positioning it as far more than a palliative agent (Panthi et al., 2023; Manginstar et al., 2024).

Quercetin’s antioxidant activity operates on two fronts: it directly neutralizes reactive oxygen and nitrogen species (ROS and RNS), which are responsible for secondary tissue damage following thermal injury, and it indirectly activates the Nrf2–ARE signaling pathway, leading to the upregulation of key antioxidant enzymes such as superoxide dismutase (SOD), catalase (CAT), and glutathione peroxidase (GPx) (Panthi et al., 2023; Priyadarshi et al., 2022; Widiatmoko et al., 2024). This redox-balancing effect is essential for initiating effective tissue repair.

Simultaneously, quercetin suppresses excessive inflammation by inhibiting central immune signaling pathways, including NF-κB and MAPK. This results in the downregulation of pro-inflammatory cytokines (TNF-α, IL-1β, IL-6) and promotes macrophage polarization toward the M2 phenotype, facilitating the transition from inflammation to regeneration (Hajam et al., 2025; El-Sherbeni and Negm, 2023). During the proliferative phase, quercetin stimulates angiogenesis via VEGF upregulation, ensuring adequate oxygen and nutrient supply to the wound bed (Kumar et al., 2023; Zhu et al., 2024). It also enhances fibroblast activity and TGF-β1 signaling, leading to robust extracellular matrix deposition and improved collagen organization (Arghand et al., 2024) timicrobial and antibiofilm properties against common burn pathogens such as *Staphylococcus aureus* and *Pseudomonas aeruginosa*, offering critical protection against infection-a major contributor to burn-related morbidity and mortality (Du et al., 2025; Shubina et al., 2024). This synergistic combination of antioxidant, anti-inflammatory, pro-angiogenic, pro-fibrotic, and antimicrobial actions-delivered by a single molecule-sets quercetin apart from conventional single-target therapies like silver sulfadiazine, which may even delay healing due to cytotoxicity (Alberts et al., 2025).

However, translating this promise into clinical practice requires a strategic and multidisciplinary research effort. The first priority is to conduct comprehensive pharmacokinetic and toxicological evaluations of optimized topical quercetin formulations. Although quercetin is considered safe in dietary use, its behavior on damaged skin remains poorly understood. Studies must address absorption, distribution, metabolism, and elimination in burn wounds, using advanced analytical techniques to assess systemic exposure and long-term safety (Utpal et al., 2025; Zaborowski et al., 2024; Lee and Kim, 2022).

Equally critical is the continued development of advanced drug delivery systems. The future of quercetin therapy lies in second-generation formulations such as nanostructured lipid carriers (NLCs), liposomes, and polymeric nanoparticles, which enhance drug stability, loading capacity, and controlled release (Bhati et al., 2024; Bhuyan et al., 2024; Rattanachot et al., 2025; Xiong et al., 2024). Research should also explore smart hydrogels that respond to wound-specific stimuli-such as pH shifts or elevated matrix metalloproteinases (MMPs)- to release quercetin precisely when and where it is needed.

The final and most decisive step is the initiation of randomized, double-blind, placebo-controlled clinical trials in human burn patients. Phase I/II trials should establish safety, tolerability, and optimal dosing regimens across different burn depths. Phase III trials must be adequately powered to compare optimized quercetin formulations against current standards of care, with clinically meaningful endpoints such as wound closure rate, time to re-epithelialization, pain reduction, scar quality (e.g., Vancouver Scar Scale), and patient satisfaction (Raheem et al., 2025; Guan et al., 2025; Chen et al., 2022). Health economic analyses should also be included to demonstrate cost-effectiveness and support adoption by healthcare systems.

Beyond monotherapy, combination strategies offer exciting potential. Quercetin may be paired with other bioactive compounds-such as curcumin for enhanced anti-inflammatory effects or Manuka honey for synergistic antimicrobial and debridement benefits. Given the global challenge of antimicrobial resistance, investigating quercetin’s ability to disrupt biofilms and potentiate antibiotic efficacy is especially urgent (Shivgotra et al., 2025). Preclinical evidence suggests that quercetin can enhance the action of conventional antibiotics against resistant strains, potentially leading to a new generation of topical therapies for infected burn wounds.

While most studies report favorable outcomes, some investigations have shown limited efficacy or formulation-dependent variability, indicating that therapeutic success is strongly influenced by delivery strategy, dosage, and experimental model.

Future studies to be conducted should be aimed at dose-response maximization, safety testing on a long-term basis, and pharmacokinetic profiling to explain therapeutic dose levels and systemic exposure patterns. Moreover, topical bioavailability and therapeutic retention at the wound site could be enhanced by nanocarrier-based formulation-based studies, lipid or hydrogel delivery system-based formulation-based studies. There would also be a value of comparative studies of standard burn treatments to establish the relative clinical efficacy. The need to extrapolate the research to large-animal models and controlled clinical trials will eventually be required to determine the translational applicability, as well as, whether the molecular mechanisms identified in this study have similar efficacy in the physiology of human burns.

Overall, this paper presents a combined body of biochemical, histological, and molecular data on the basis of the therapeutic value of quercetin in burn wounds healing. The findings are all warning signs that its positive actions are due to the orchestration of changes in oxidative stress, inflammatory signaling, and tissue regeneration-signaling, in place of one mechanism. Such an integrated activity profile increases its therapeutic usefulness and fosters additional examination to the clinical translation.

Combined, these results place quercetin as a prospective agent in the adjunctive therapy of burns, which requires further pharmacological optimization and testing on sophisticated experimental and clinical systems.
